# RAGE and TGF-β1 Cross-Talk Regulate Extracellular Matrix Turnover and Cytokine Synthesis in AGEs Exposed Fibroblast Cells

**DOI:** 10.1371/journal.pone.0152376

**Published:** 2016-03-25

**Authors:** Andreea Iren Serban, Loredana Stanca, Ovidiu Ionut Geicu, Maria Cristina Munteanu, Anca Dinischiotu

**Affiliations:** 1 Department of Preclinical Sciences, University of Agronomical Sciences and Veterinary Medicine, 105 Splaiul Independentei, 050097, Bucharest, Romania; 2 Department of Biochemistry and Molecular Biology, University of Bucharest, 91–95 Splaiul Independentei, 050095, Bucharest, Romania; 3 Department of Physiological Sciences, Oklahoma State University, 264 McElroy Hall, Stillwater, Oklahoma, 74078, United States of America; University of Oklahoma Health Sciences Center, UNITED STATES

## Abstract

AGEs accumulation in the skin affects extracellular matrix (ECM) turnover and triggers diabetes associated skin conditions and accelerated skin aging. The receptor of AGEs (RAGE) has an essential contribution to cellular dysfunction driven by chronic inflammatory responses while TGF-β1 is critical in both dermal homeostasis and inflammation. We investigated the contribution of RAGE and TGF-β1 to the modulation of inflammatory response and ECM turnover in AGEs milieu, using a normal fibroblast cell line. RAGE, TGF-β1, collagen I and III gene and protein expression were upregulated after exposure to AGEs-BSA, and MMP-2 was activated. AGEs-RAGE was pivotal in NF-κB dependent collagen I expression and joined with TGF-β1 to stimulate collagen III expression, probably via ERK1/2 signaling. AGEs-RAGE axis induced upregulation of TGF-β1, TNF-α and IL-8 cytokines. TNF-α and IL-8 were subjected to TGF-β1 negative regulation. RAGE’s proinflammatory signaling also antagonized AGEs-TGF-β1 induced fibroblast contraction, suggesting the existence of an inhibitory cross-talk mechanism between TGF-β1 and RAGE signaling. RAGE and TGF-β1 stimulated anti-inflammatory cytokines IL-2 and IL-4 expression. GM-CSF and IL-6 expression appeared to be dependent only on TGF-β1 signaling. Our data also indicated that IFN-γ upregulated in AGEs-BSA milieu in a RAGE and TGF-β1 independent mechanism. Our findings raise the possibility that RAGE and TGF-β1 are both involved in fibrosis development in a complex cross-talk mechanism, while also acting on their own individual targets. This study contributes to the understanding of impaired wound healing associated with diabetes complications.

## Introduction

Advanced glycation end products (AGEs) are formed by a non-enzymatic glycosylation reaction named glycation or Maillard reaction. Glycation was first described by Maillard in 1912 as a process involved in food browning during thermal processing. Maillard also hypothesized that glycation could play a role in diabetes [1]. More than 50 years later, Bookchin and Gallop had discovered the first glycated protein (glycated hemoglobin) in diabetes patients [2]. Since then, the presence of glycation products in living systems and their involvement in diabetes pathologies and aging have been an intensive field of research. The glycation reaction begins with the attack of the aldehyde group of reducing sugars on the free amino residue of proteins resulting in the formation of a Schiff's base, which spontaneously rearranges into an Amadori's product. In time, these early glycation products undergo further reactions such as rearrangements, dehydrations, oxidations and condensations that ultimately result in the development of heterogeneous structure compounds such as *N*-ε-carboxy-ethyl-lysine, pentosidine, glucosepane, glyoxal-lysine dimers and methyl-glyoxal-lysine dimers, collectively known as advanced glycation end products (AGEs). These compounds are increasingly accumulated in aging and diabetic skin [3–6]. Thus, skin aging could be viewed as a life-time accumulation of AGEs, which may be accelerated under diabetic circumstances. Enhanced formation and crosslinking of AGEs on other macromolecules [7], such as collagen or other long life proteins [8–10], renders them resistant to turnover [11]. These altered substrates not only contribute to ECM hypertrophy by their gradual accumulation but also act as agonists for a series of surface receptors. Along with scavenger receptors shown to bind AGEs, several AGEs receptors were identified (RAGE, AGE-R1/OST-48, AGE-R2/80K-H, AGE-R3/galectin-3) [12]. Additionally, AGEs were shown to induce effects via nonspecific mechanisms [13]. To date, AGEs/RAGE activation has been associated with maladaptive ECM alterations leading to increased vascular permeability, contractility, ECM synthesis, cell growth, perturbed cell-matrix interactions, altered cell adhesion and apoptosis [14]. Chronic exposure to AGEs is also known to induce maladaptations of the immune response, which are thought to be responsible for the plethora of immune-related diabetes complications, including difficulty in skin healing and a predisposition to infections [15,16].

Fibroblast cells are ubiquitously expressed in an organism, as they are responsible for ECM synthesis and remodeling. Moreover, fibroblasts have dynamic roles in the immune response, sensing potential dangers, dispatching chemokines and cytokines to invoke the immune cells, and directing the growth of surrounding specialized cells, by secreting growth factors and modulating the composition of the ECM [17]. Both these aspects are very important in wound healing, which is an important issue with diabetic patients [11,15,16,18]. Fibroblasts exert mechanical forces upon the surrounding extracellular matrix, which lead to wound contraction and closure [19], albeit, prolonged fibroblast contraction is a maladaptative process, and a hallmark of fibrosis [18,20]. Interestingly, diabetes complications have been associated with increased fibrogenesis [21–23] brought upon by increased active TGF-β1 levels [24]. Nonetheless, reports have shown that glycated collagen matrices are not properly processed by fibroblasts [11,25], which is thought to be responsible for the delayed or impaired wound healing in diabetes complications.

Findings which emphasized that RAGE receptor blockade or inhibition of RAGE’s transcriptional and/or translational expression reduce inflammation and atherosclerosis have intimately linked AGEs to RAGE, which is currently viewed as the central mediator and amplifier of the processes initiated by AGEs. A very compelling body of literature has emphasized the link between a cell’s ability to express RAGE and its capacity to respond to AGEs [26,27]. Therefore, the AGEs/RAGE signaling is pivotal for the understanding and efficient therapy of diabetes-mediated ECM related diseases.

The aberrant and chronic immune responses observed in many diabetes complications, are due, in part to altered ECM composition and structure. AGEs involvement in skin aging has become evident in recent years, as they were shown to induce or maintain pro-inflammatory changes by activation of matrix metalloproteinases (MMPs) and proinflammatory cytokine production, which contribute to many structural changes observed in aged skin [3,28].

It is well established that one consequence of RAGE activation is the upregulation of RAGE itself, through a NF-κB dependent mechanism [29,30]. TGF-β1 growth factor is subjected to NF-κB transcriptional activation as well (not excluding other transcriptional factors) [31], although RAGE and TGF-β1 regulation is controlled by a number of shared transcriptional factors [30,31]. Both AGEs/RAGE and TGF-β1 signaling have been demonstrated to activate extracellular signal-regulated kinases (ERK) [32–34]. The ERKs subfamily of MAPKs are responsible for multiple cellular functions, such as differentiation and proliferation [35]. Importantly, ERK signaling was shown to be essential for fibroblast differentiation [34] and fibroblast activation [24], resulting in increased matrix production and accumulation, reduced cell migration, and greater contractility. However, RAGE signaling is generally considered to be pro-inflammatory and anti-fibrotic [36], while TGF-β1 is pro-fibrotic and anti-inflammatory [37,38]. Since increased AGEs exposure characteristic of diabetes was shown to induce both signaling cascades, the effect of concurrent RAGE and TGF-β1 signaling outcome in fibrogenesis-related diabetes complications is not fully elucidated.

The effects of AGEs exposure in fibroblasts merit increased attention and, we aimed at differentiating the contributions of RAGE and TGF-β1 growth factor in eliciting AGEs induced responses using specific blocking antibodies, in order to understand the development of fibrosis complications and accelerated skin aging associated with diabetes.

We highlighted that a 24 h exposure to AGEs-BSA induced in normal fibroblast cells a protein expression profile similar to the one of aging cells, characterized by increased active TGF-β1 and MMP-2 activity [39] and reduced expression of collagen type I [40]. CCD-1070Sk fibroblasts stimulation with AGEs-BSA induced a decrease of the relative gene expression ratios of collagen I/III compared to the gene expression registered after 12 h of exposure, possibly contributing to a future imbalance in collagen I/III protein expression ratio. In this study we showed that collagen type I synthesis was diminished by RAGE blockade, while collagen III expression was diminished by both RAGE and TGF-β1 blocking antibodies. Importantly, AGEs-induced upregulation of IL-8 and TNF-α cytokines was dependent on RAGE signaling and inhibited by TGF-β1, suggesting the existence of paradoxical consequences of RAGE and TGF-β1 activation.

## Materials and Methods

### AGEs-BSA preparation, characterization and quantification

The glycated BSA preparation method, the degree of BSA modification after glycation and AGEs quantification were previously described [41]. Briefly, endotoxin-free fraction V of bovine serum albumin (RIA grade) was incubated in PBS (free of trace metal ions), under sterile conditions with 0.5 M *D*-glucose for 3 months at 37°C. After the unreacted glucose was removed, the BSA molecular weight was assessed by size exclusion chromatography and SDS–PAGE, revealing that glycated BSA (AGEs-BSA) monomers increased in molecular weight by ~8–10 kDa compared to BSA monomers. A tendency to form dimers was also observed in AGEs-BSA [41]. The characteristic fluorescence emission spectra of glycation products were assessed at λEx/Em = 335 nm/ 385 nm (pentosidine fluorescence) and λEx/Em = 340 nm/ 370 nm (AGEs fluorescence) and revealed significant increases at both excitation/emission wavelengths by 3 and 2.25 fold compared to unmodified BSA. The total AGEs content was evaluated using an ELISA assay (Advanced Glycation End Product ELISA Kit, Cell Biolabs, San Diego, CA, USA) which revealed that the glycated products content in AGEs-BSA increased by 3.2 fold compared to BSA, reaching 4.8 ng/μg protein [42].

### Cell culture and treatment

CCD-1070Sk (ATCC^®^ CRL-2091) cells were grown in EMEM medium (Life Technologies, Carlsbad, CA, USA), supplemented with 1% antibiotic antimycotic solution, 1.0 mm sodium pyruvate, 0.1 mM non essential amino acids, 1.5 g/L sodium bicarbonate and 10% fetal bovine serum in 5% CO_2_ air atmosphere at 37°C. The cultured cells were gradually acclimatized to the serum free environment by decreasing the fetal bovine serum concentration. After the cultures were synchronized, cells were treated for 12 h and 24 h with 50, 100 and 200 μg/ml AGEs-BSA or BSA as control. Also, the cells were subjected for 24 h to a co-treatment with 200 μg/ml AGEs-BSA and 20 ng/ml anti-RAGE or anti-TGF-β1 antibodies, and 200 μg/ml AGEs-BSA or BSA and non-immune mouse IgG (MB Biomedicals, Solon, OH, USA) as controls. Cell viability was evaluated using the TC20 automated cell counter (Bio-Rad Laboratories, Hercules, CA, USA).

### Conditional media preparation

At the end of each treatment interval, culture supernatants were harvested and centrifuged to remove cell debris, and then concentrated using the 3 kDa cut off membrane (Millipore, St. Charles, MO, USA). Protein concentration was assessed using Bradford's method [43]. The conditioned media samples were aliquoted and stored at −80°C.

### Membrane protein fractions isolation

Cell membrane fractions were obtained using the RedyPrep Protein Extraction Kit (Bio-Rad Laboratories, Hercules, CA, USA) according to manufacturer’s instructions, and modified as previously described [41,42].

### Quantification of Inflammatory Cytokines and TGF-β1

Conditional media samples were used for the detection of secreted IL-2, IL-4, IL-6, IL-8, IL-10, GM-CSF, IFN-γ and TNF-α cytokines as previously described [42], using the Bio-Plex Pro Human Cytokine 8-plex panel (Bio-Rad Laboratories, Hercules, CA, USA)., The bioactive TGF-β1 concentrations from conditional media samples were determined using the TGF-β1 Emax ImmunoAssay System ELISA kit (Promega Co., Madison, WI, USA), according to manufacturer’s instructions.

### Gelatin zymography

Metalloproteinase gelatinolytic activity from concentrated conditional media samples (12.5 μg/well total protein) was assessed by polyacrylamide gel electrophoresis (PAGE) in 7.5% polyacrylamide gels containing gelatin, in non-reducing conditions. Gelatinolytic bands were observed after overnight gel’s incubation in 1M Tris-HCl buffer, pH 7.6 containing 10 mM CaCl_2_, at 37°C and subsequent staining with Coomassie Brilliant Blue R-250 as described previously [41].

### Collagen contraction assay

The assay was done according to the protocol described by Ngo *et al*. [44]. Cultured cells were subjected to the 24 h co-treatment with 200 μg/ml AGEs-BSA and 20 ng/ml anti-RAGE or anti-TGF-β1 antibodies, and 200 μg/ml AGEs-BSA or BSA and non-immune mouse IgG as controls. After 24 h, cells were detached using trypsin, counted, suspended in 300 μl growth medium and mixed with 150 μl 3% collagen solution in 1% acetic acid, and immediately the mix was neutralized with 1M NaOH. Final cell density was 1.5×10^5^ cells/ml. After collagen gelation, the gels were covered with 1 ml complete culture medium and incubated in the cell house over night. The gels were photographed with a Canon EOS 600D camera, with a 90 mm macro lens. Gel areas were calculated using the Image J software.

### Western Blot assays

Proteins from conditional media and from purified membrane protein fraction were denatured and separated by PAGE under reducing conditions followed by transfer to PVDF membrane. Membranes were blocked (Western Breeze Chromogenic Immunodetection Kit, Life Technologies, Carlsbad, CA, USA) and then incubated with primary antibodies: anti-RAGE (RD9C 2, Santa Cruz Biotehnology), anti-TGF-β1 (9016.2, Santa Cruz Biotehnology), anti-collagen I (I-8H5, MP Biomedicals, Solon, OH, USA), anti-collagen III (III-53, MP Biomedicals, Solon, OH, USA) and rabbit anti-MMP-2 (M4065, Sigma-Aldrich, Saint Luis, Missouri, USA). Alkaline-phosphatase conjugated secondary antibodies were used. Immunoreactive bands were developed with the Western Breeze Chromogenic Immunodetection Kits (Life Technologies, Carlsbad, CA, USA) and quantified with BioCapt 12.6 software (Vilbert Lourmat, France). The expression of target proteins was normalized against the total proteins blotted onto PVDF membranes (for the corresponding lane) stained with Ponceau S solution (before blocking the electroblotted membranes), as total protein loading control [45].

For NF-κB p65 and phosphorilated ERK1/2 detection, whole-cell protein extracts were resolved on Mini-PROTEAN TGX Stain Free 4%–15% precast gels (Bio-Rad Laboratories, Hercules, CA, USA), transferred onto 2 μm PDVF (V3 Western Workflow, Bio-Rad Laboratories, Hercules, CA, USA) and digitalized using the ChemiDoc MP System (Bio-Rad Laboratories). Total proteins transferred were quantified using the Image Lab software (version 5.0, Bio-Rad Laboratories) and the membranes were blocked using 5% non-fat dry milk, overnight. Polyclonal NF-κB p65 (VPA00015), mouse anti-human ERK/MAPK (pThr202/pTyr204) (F04-4G10), HRP conjugated secondary antibody (5184–2504) from AbD Serotec, Oxford, UK were used, and blots were revealed using the Clarity Western ECL Substrate (Bio-Rad Laboratories, Hercules, CA, USA). The chemiluminescence signal was detected using the ChemiDoc MP System, driven by the Image Lab software (version 5.2.1). The NF-κB p65 and p-ERK1/2 protein expression was quantified using the Image Lab software, and normalized to the total proteins transferred onto the membrane (each protein band was normalized against the total proteins transferred in the corresponding lane).

### Semiquantitative Real-time RT-PCR

Total RNA extraction from cultured cells was performed using TRIzol reagent (Life Technologies, Carlsbad, CA, USA) after Chomezynski's modified method [46]. The concentration and purity of the extracted total RNA were assessed using the 260/280 nm absorbance ratio. The integrity of total RNA was verified using the 2100 Bioanalyzer platform (Agilent, Santa Clara, CA, USA) and the 6000 RNA Nano Kit (Agilent, Santa Clara, CA, USA), following the standard procedures.

Bio-Rad iScript cDNA synthesis Kit (Bio-Rad Laboratories, Hercules, CA, USA) was used to reverse transcribe the total RNA into cDNA, according to the manufacturer’s instructions. The specific sense and antisense primers were designed using the NCBI Database and Primer3 Input software (version 0.4.0) (Table 1). *GAPDH* was used as reference gene.

**Table 1:** The target genes and the primer sequences used for semi quantitative real-time RT- PCR.

*Gene*/mRNA reference sequence	Oligonucleotide primer sequences (5'-3')	Annealing temperatures (°C)
*Procollagen 1 α1*/NM_001845.4	**F**: GACGGCTTACCTGGAGAC; **R**: GGGAAGACCTGGCAAACC	58
*Procollagen 3 α1/*NM_000090.3	**F**: GGAGTAGCAGTAGGAGGAC; **R**: AACCAGGATGACCAGATGTA	55
*RAGE/*NM_001136.4	**F**: TGGATGAAGGATGGTGTG; **R**: GATGATGCTGATGCTGAC	49
*TGF-β1/*NM_000660.4	**F**: GACACCAACTATTGCTTCAG; **R**: CAGGCTCCAAATGTAGGG	55
*GAPDH/*NM_002046.4	**F**: TGGTCTCCTCTGACTTCAAC; **R**:GTGAGGGTCTCTCTCTTCCT	58

Real-time semi-quantitative RT-PCR was performed using SYBR Green Supermix Kit (Bio-Rad Laboratories, Hercules, CA, USA) and 200 nM of each forward and reverse primers. All PCR amplification reactions were realized using the BioRad iCycler iQ. Melting curve analysis showed a single product for each gene transcript. The target genes relative expression were calculated according to Pfaffl method [47].

### Statistical analysis

Unless mentioned otherwise, all data represent the mean ± SD from three independent experiments. An unpaired two-tailed Student’s t test, unequal distribution, was used to determine the significant changes between two groups of data. A statistically significant *p* value *p*<0.05 was noted *, while *p*<0.01 and *p*<0.001 were indicated by ** and *** respectively.

## Results and Discussions

**Fig 1:**
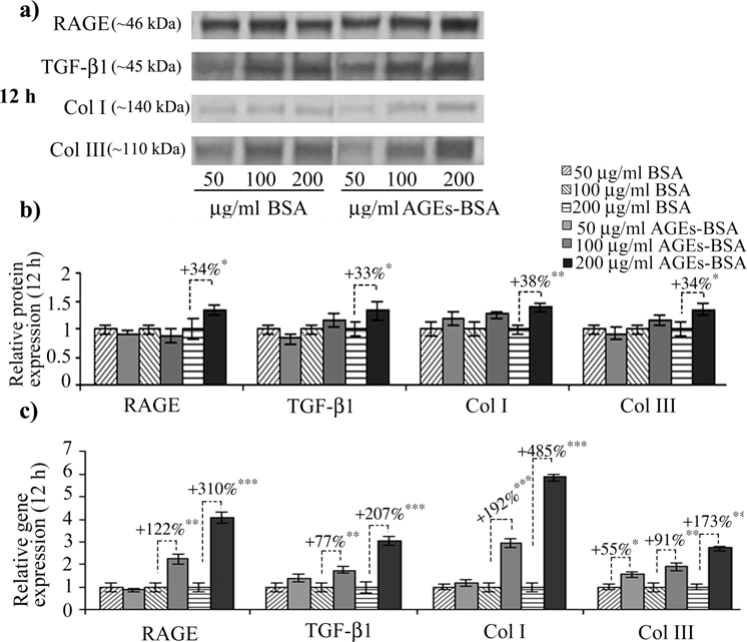
Increased RAGE, latent TGF-β1, collagen I and III gene and protein expression in CCD-1070Sk fibroblasts after 12 h of AGE-BSA exposure. Representative immunoblots (a) and the corresponding densitometry analysis. Each immunoreactive band was normalized to the total proteins transferred in the corresponding lane. Data are relative to controls (BSA treated cells) and represent means ± SD (b). Relative gene expression for RAGE, TGF-β1, collagen (Col) I and III levels is shown in (c). *p* values indicate statistically significant changes * for *p*< 0.05; ** for *p*< 0.01; *** for *p*< 0.001.

**Fig 2:**
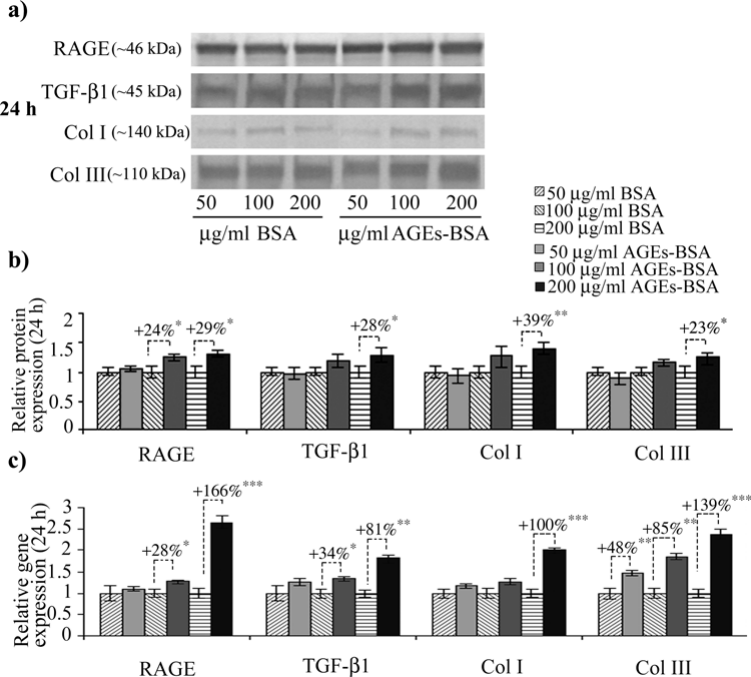
Increased RAGE, latent TGF-β1, collagen I and III gene and protein expression in CCD-1070Sk fibroblasts after 24 h of AGE-BSA exposure. Representative immunoblots (a) and the corresponding densitometry analysis. Each immunoreactive band was normalized to the total proteins transferred in the corresponding lane. Data are relative to controls (BSA treated cells) and represent means ± SD (b). Relative gene expression for RAGE, TGF-β1, Col I and III levels is shown in (c). *p* values indicate statistically significant changes * for *p*< 0.05; ** for *p*< 0.01; *** for *p*< 0.001.

**Fig 3:**
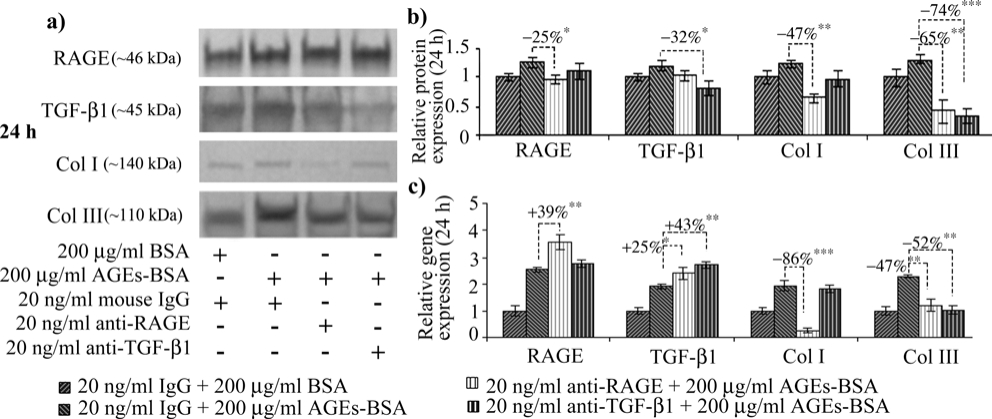
The effects of RAGE or TGF-β1 antibody blockade in CCD-1070Sk cells exposed for 24 h to 200 μg/ml AGEs-BSA. Representative immunoblots for RAGE, TGF-β1, Col I and III (a) and the corresponding densitometry analysis. Each immunoreactive band was normalized to the total proteins transferred in the corresponding lane. Data are relative to controls (BSA treated cells) and represent means ± SD (b). Relative gene expression of RAGE, TGF-β1, Col I and III is shown in (c). *p* values indicate statistically significant changes * *p*< 0.05; ** *p*< 0.01; *** *p*< 0.001.

We noted a high basal-level of RAGE expression in CCD-1070Sk normal skin fibroblasts, an observation concurring with other studies on RAGE expression in normal non-diabetic fibroblasts [48]. After 12 h of exposure to 200 μg/ml AGEs-BSA, RAGE protein expression increased (Fig 1A and 1B); a consistent increase was also observed in RAGE’s gene expression levels, which increased after the treatment with 100 and 200 μg/ml AGEs-BSA doses, by over 2 and 4-fold the expression observed in control cells exposed to BSA (Fig 1C). After 24 h, RAGE protein level was significantly increased in cells exposed to 100 and 200 μg/ml AGEs (Fig 2A and 2B), while the gene expression was increased by a lesser extent compared to the 12 h interval. An unexpected finding was that RAGE blockade with anti-RAGE antibodies during the 24 h exposure to 200 μg/ml AGEs-BSA dose diminished the receptor’s protein level down to the level of BSA exposed cells (Fig 3A and 3B) as opposed to the gene expression, which was additionally increased (Fig 3C). In this context, we could assume that an exposure to anti-RAGE antibodies longer than 24 h may have a RAGE agonistic effect, by stimulating *de novo* expression of the receptor.

The 12 h exposure to 100 μg/ml AGEs-BSA dose induced TGF-β1 protein expression (Fig 1), while the 200 μg/ml AGEs-BSA dose increased both the gene and protein expression of TGF-β1 (Fig 1C). After 24 h, the intensity of TGF-β1 upregulation was diminished compared with the 12 h exposure (Fig 2), nevertheless the protein and mRNA expression remained significantly higher than those corresponding to control cells. One important aspect of TGF-β1 pro-fibrotic effects is increased collagen I synthesis, and signaling pathway activated by TGF-β1 leading to collagen I synthesis was recently described [49]. This mechanism involves two different molecular pathways for collagen I upregulation, one activated in the first few hours following TGF-β1 exposure (TGF-βRI and TGF-βRII heterodimerization is followed by Smad2/3 phosphorilation which subsequently form a complex with Smad4, translocates into the nucleus and drives collagen transcription) and the other one acting after an exposure longer than 12 h (after TM4SF20 depletion, CREB3L1 is cleaved by S1P and S2P, releasing the NH_2_-terminal domain which forms a complex with Smad4 and activates collagen gene transcription). The former mechanism was recently revealed to be necessary for sustained TGF-β1-induced collagen synthesis [49]. The same mechanism responsible for collagen upregulation in chronic exposure to TGF-β1 is also involved in the transcriptional inhibition of genes involved in cell proliferation [50], however, in our experimental setup, cell viability was not affected (data not shown). Recent evidence has indicated CREB3L1 mRNA and protein expression were not detectable in fibroblast cell types [51]. This would explain why the fold increase of collagen I gene expression were smaller after the 24 h AGEs-BSA exposure compared to the 12 h interval (Figs 1C and 2C).

Notably, after 24 h, the increases of collagen III gene and protein expression exceeded those of collagen I (Fig 2). A recent study has shown that fibroblast cells stimulated by conditioned media rich in connective tissue growth factor collected from TGF-β1 treated smooth muscle cells demonstrated a preference to synthesize collage III in disfavor of collagen I [52]. Fibroblasts expressing high levels of collagen III were also isolated from intestinal strictures of Chrone’s disease patients, a characteristic not shared with fibroblasts from inflamed tissues [53]. Diminished collagen I deposition is a hallmark of aged skin [40] and also diabetes associated skin disorders [54]. The immunoblot quantitative analysis revealed that collagen I protein expression did not increase significantly between the two AGEs-BSA exposure intervals, although very consistent *procollagen I* mRNA levels were detected after 12 h. This situation might be explained by the elevated MMP-2 expression and activity registered at this AGEs-BSA exposure interval (Fig 3). MMP-2 has a distinct ability to degrade type-I collagen, a major component of the basement membrane and extracellular matrix [55,56], while also being actively involved in the modulation of inflammatory response [57]. The reversal of the collagen I to collagen III gene expression ratio we reported after 24 h of exposure to 200 μg/ml AGEs-BSA could be of significance in an *in vivo* context, as they could result in altered collagen I vs. collagen III deposition in the ECM structure, changes associated with reduced tensile strength and elasticity rendering skin more prone to injury [58], and impaired wound healing in diabetes [54].

**Fig 4:**
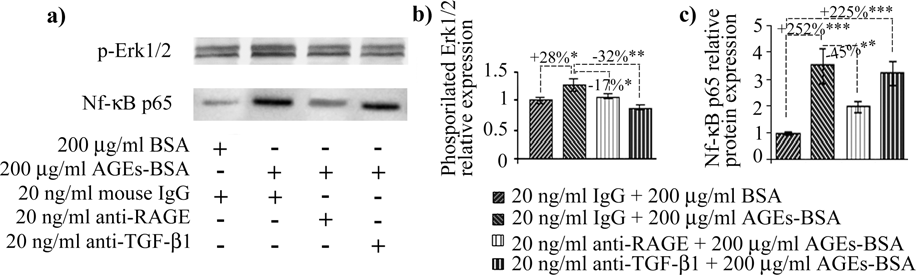
AGEs induced signaling in CCD 1070SK fibroblasts. p-ERK1/2 and NF-κB p65 protein expression levels (a) Representative immunoblot membranes; (b) Combined densitometry data indicating fold changes in the protein expression of p-ERK1/2 (b) and NF-κB p65 (c). Immunoreactive bands were normalized to the total proteins transferred onto the membrane in the corresponding lane. Data are relative to BSA and nonimmune IgG treated cells. BSA: bovine serum albumin; AGEs: advanced glycation end products; * p < 0.05; ** p < 0.01; *** p < 0.001.

RAGE antibody treatment heavily impacted the collagen protein and gene expression. Most importantly, collagen I diminished to a level below the one registered in BSA and nonimmune murine IgG exposed cells, suggesting RAGE may be involved (via NF-κB signaling) in steady state collagen I expression in these cells. Recently, collagen type I expression was shown to be induced in a very specific manner by NF-κB p65 via the AGEs-RAGE axis [59]. This is in accordance with our findings which showed that anti-RAGE antibody treatment severely diminished NF-κB p65 (Fig 4). This result upholds the findings from other studies, which indicated sustained collagen I upregulation induced via TGF-β1 signaling is dependent on CREB3L1 [49], a protein not expressed by fibroblast cells [51]. In these conditions, we advance the idea that collagen I synthesis is induced via a RAGE-dependent mechanism, and TGF-β1 is probably not involved in this ECM component synthesis in fibroblasts exposed to AGEs-BSA, after an exposure interval of 24 h.

Fibroblasts exposed for 24 h to 200 μg/ml AGEs-BSA and treated with anti-RAGE antibodies had also induced an additional up-regulation of TGF-β1 mRNA, surpassing the effect of AGEs-BSA alone (Fig 3C). TGF-β1 is known to be induced by many pathways [31], including RAGE–NF-κB signaling [60]. We presume that RAGE blockade might have induced a temporary deficiency in TGF-β1 levels, a situation the cells compensate by increasing TGF-β1 gene expression via other signaling pathways [31]. Similarly, the protein levels of latent TGF-β1 decreased in AGEs-BSA exposed cells treated with anti-TGF-β1 antibodies (Fig 3A and 3B). The gene expression however, registered an additional increase compared to AGEs-BSA exposed cells (Fig 3C), as the cells overcompensated the cytokine’s depletion.

Both RAGE and TGF-β1 antibody blockade reduced collagen III expression (Fig 3), and diminished the amount of p-ERK1/2, compared to AGEs-BSA exposed cells (Fig 4). This could suggest that ERK signaling, known to increase matrix production, is in our case, involved in collagen III production during joined RAGE and TGF-β1 stimulation induced via AGEs. These results are in agreement with a study of Xu *et al*., which showed that S100A9, a ligand of RAGE, activated ERK signaling in order to drive collagen III transcriptional expression [61]. Many studies have demonstrated that TGF-β1-induced fibroblast activation requires ERK signaling [24,32,33,62], however, this is the first report showing that collagen III expression is dependent on RAGE and TGF-β1 collective signaling, linked at ERK1/2 level.

AGEs contribution to skin aging and chronic inflammation is also exerted via the modulation of MMPs activity which contribute to the structural changes observed in aged and diabetic skin. Gelatin zymography revealed only bands corresponding to pro-MMP-2 and active MMP-2 activity, while MMP-9 was not detected, a particularity of fibroblast cells, also described by others [63]. In general, MMPs are known to be down-regulated by TGF-β1, with the important exception of MMP-2 and -9 [64].

**Fig 5:**
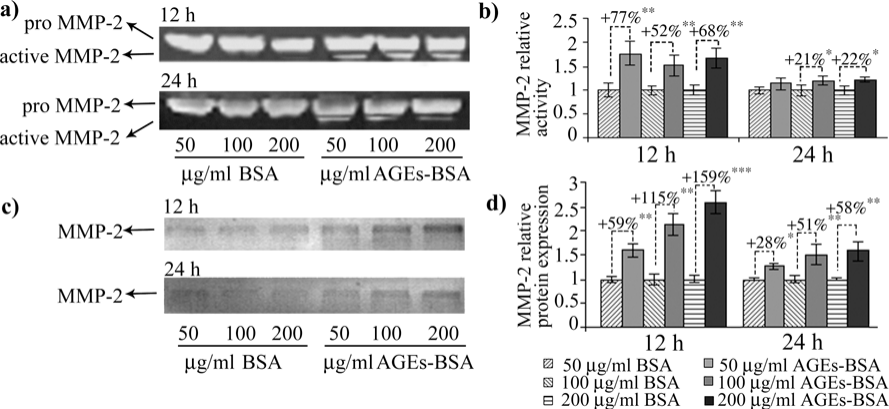
MMP-2 activity and protein expression levels in AGEs-BSA exposed CCD-1070Sk cells. MMP-2 gelatinolytic bands after 12 h and 24 h of AGEs exposure (a). Densitometry analysis of gelatinolytic bands is shown in (b). MMP-2 immunoreactive protein bands after 12 h and 24 h AGEs exposure (c). Densitometry analysis of MMP-2 immunoreactive bands is shown in (d). Densitometry data represent means ± SD. * *p*< 0.05; ** *p*< 0.01; *** *p*< 0.001.

**Fig 6:**
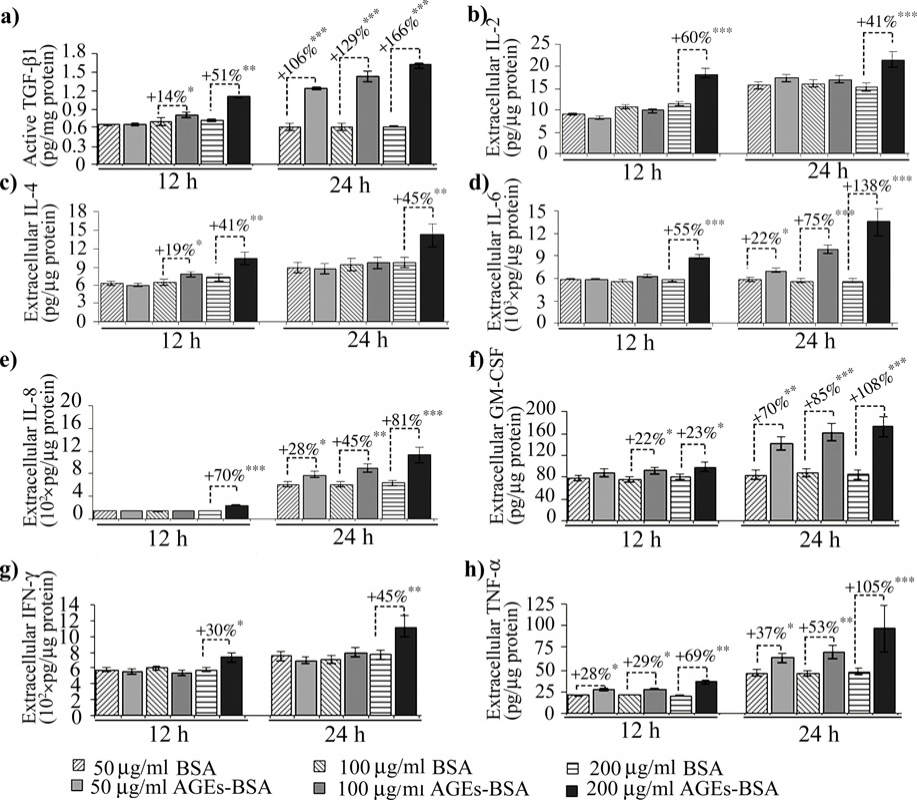
Cytokine levels in the cell culture media of CCD-1070Sk cells after 12 or 24 h exposure to 50, 100 or 200 μg/ml AGEs-BSA. Data represent absolute values ± SD. Statistically significant changes are indicated * *p*< 0.05; ** *p*< 0.01; *** *p*< 0.001.

**Fig 7:**
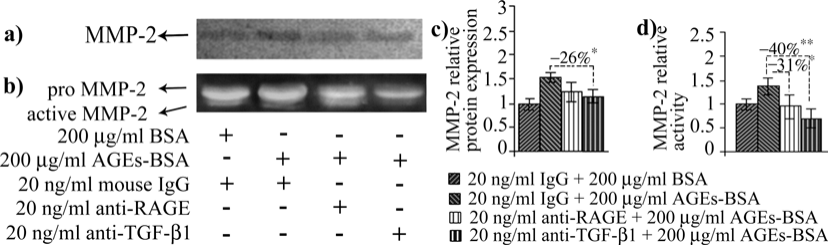
**The effects of RAGE or TGF-β1 on MMP-2** protein expression (a) and activity (b). Densitometry analysis for the immunoblot is shown in (c) and for the zymogram in (d). Densitometry data represent means ± SD. * *p*< 0.05; ** *p*< 0.01; *** *p*< 0.001.

**Fig 8:**
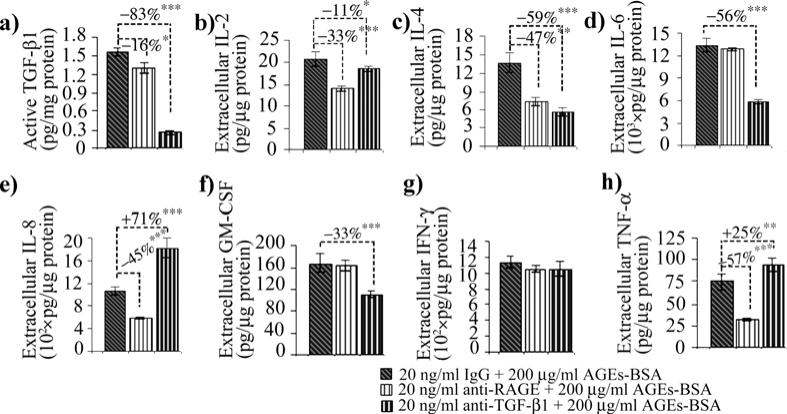
Active TGF-β1 and cytokine levels in the cell culture media of CCD-1070Sk cells after RAGE or TGF-β1 blockade after 24 h exposure to 200 μg/ml AGEs-BSA. Data represent absolute values ± SD. Statistically significant changes are indicated * p< 0.05; ** p< 0.01; *** p< 0.001.

In our experimental conditions, MMP-2 activity increased after 12 h of AGEs-BSA exposure at all doses, while its protein expression was induced in a dose dependent manner, with a maximum of over 2.5 fold increase after the 12 h exposure to 200 μg/ml AGEs-BSA (Fig 5). Surprisingly, the highest activity and expression levels were registered in the first time interval analyzed. The more moderate increases in activity and protein expression reported after 24 h of AGEs-BSA exposure could be associated with the increase of IFN γ and TNF-α cytokines (Fig 6G and 6H), which were shown to possess inhibitory effects on MMP-2 expression [65]. Decreased RAGE expression or RAGE inhibition were also shown to negatively impact on MMP-2 expression and activity [66,67]. This could be a possible explanation for the more modest MMP-2 activity registered after 24 h, considering that RAGE expression is also moderately reduced after 24 h of AGEs-BSA challenge, compared to the 12 h challenge (Figs 1 and 2). RAGE blockade reduced MMP-2 activity to control level, while TGF-β1 antibody blockade impacted both the gelatinase’s activity and protein expression (Fig 7). MMP-2 inhibition was more strongly associated with TGF-β1 blockade, as this approach surprisingly resulted in an additional upregulation of TNF-α cytokine (Fig 8H).

While the latent TGF-β1 displayed moderate expression increases after exposure to 200 μg/ml AGEs-BSA for 12 and 24 h (Figs 1A and 1B and 2A and 2B), the active form of TGF-β1 followed a more dramatic evolution and increased in a dose and time-dependent manner starting with the 12 h exposure to 100 μg/ml AGEs-BSA (Fig 6A). Previous studies in several mammalian cell types have shown that latent TGF-β1 can be activated *in vitro* by MMP-2 and by activators of MMP-2 [68,69]. *In vivo*, TGF-β1 activation was correlated with increased MMP-2 activity, associated with advancing age [39]. After 24 h of exposure to AGEs-BSA, active TGF-β1 levels further increased, probably as a result to the cytokine’s accumulation in the cell culture medium (Fig 6A). Active TGF-β1 decreased in AGEs-BSA exposed cells treated with anti-TGF-β1 antibodies reaching a level below the constitutive expression of BSA treated cells (Fig 8A).

IL-2 and IL-4 are considered anti-inflammatory cytokines [70]. Their expression increased significantly in 200 μg/ml AGEs-BSA exposed cells for 12 and 24 h (Fig 6B and 6C). Surprisingly, IL-2 levels fold increases were higher after 12 h compared to the 24 h interval. IL-4 expression was also significantly activated in 100 μg/ml AGEs-BSA exposed cells for 12 h (Fig 6C), which was lost after 24 h. This would suggest that their anti-inflammatory effects are diminished at the 24 h interval of AGEs-BSA exposure. IL-4 has been reported to either increase the expression of TGF-β1 [71] or directly stimulate fibroblasts to produce collagen [72] and have a pro-fibrotic overall effect [73]. IL-2 and IL-4 cytokine expressions were reduced by both the anti-RAGE and anti-TGF-β1 antibody treatments, suggesting both RAGE and TGF-β1 signaling pathways were activated by AGEs-BSA and converged to upregulate these cytokines (Fig 8B and 8C).

AGEs are involved in cytokine maturation and release, triggering a local inflammatory response [3,28]. IL-6 and IL-8 pro-inflammatory cytokines expression increased in the cell culture medium 12 h only after the 200 μg/ml AGEs-BSA dose, whereas after 24 h they rose in a dose-dependent manner (Fig 6D and 6E). IL-6 and IL-8 expression were shown to be induced via multiple signaling pathways activated consequently to RAGE ligand recognition, including NF-κB [74]. In a previous study on HEK 293 cells, we suggested that IL-6 cytokine is induced in AGEs-BSA milieu via a different signaling pathway, alternative to RAGE activation [42]. We have shown that IL-6 and TGF-β1 expression levels had very similar expression profiles in HEK 293 cells exposed to AGEs-BSA, being increased at an early time interval (12 h) when other cytokines were not elevated [41,42], suggesting a possible relation between IL-6 and TGF-β1. In other studies both cytokines were shown to be strongly induced in TNF-α stimulated skin fibroblasts [75,76]. Interestingly, our current data revealed that TNF-α expression was the only one to be increased starting with the 50 μg/ml AGEs-BSA dose after 12 h exposure (Fig 6H), thus possibly contributing to other cytokine upregulation, it is most probably not involved in upregulating IL-6. The anti-RAGE antibody treatment strongly inhibited NF-κB p65 (Fig 4), and TNF-α (Fig 8H) while IL-6 expression remained unchanged (Fig 8). Moreover, TGF-β1 blockade diminished IL-6 while having an opposite outcome on TNF-α levels (Fig 8).

AGEs induced TNF-α increases were associated with the activation of several signaling pathways [77]. The increased levels we reported after 12 h of 50, and 100 μg/ml AGEs-BSA treatments could probably be due to TNF-α activation by MMP-2. The mechanism of TNF-α processing and activation by MMP-2 and other MMPs *in vitro* was previously described [78]. In our case, MMP-2 demonstrated increased activity at all AGEs-BSA doses applied for 12 h, which probably contributed to TNF-α activation. The high levels of cytokine detected at the 24 h interval could be a consequence of the cytokine’s accumulation in the cell culture medium (Fig 6H), and possibly reflect the increased *de novo* synthesis induced by RAGE signaling.

**Fig 9:**
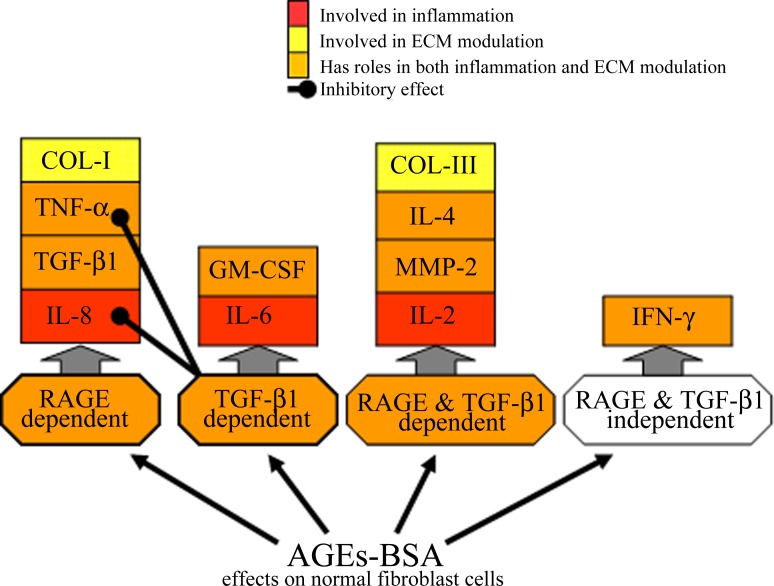
Schematic representation of the main results of our study, highlighting the factors involved in ECM modulation activated by AGEs exposure.

TNF-α was shown to induce synthesis of GM-CSF in smooth muscle cells of patients with idiopathic pulmonary fibrosis, an affliction associated with increased TGF-β1 production rather than exacerbated inflammation [79–81]. GM-CSF is associated with fibroblast proliferation and fibrosis on the one side, and with promoting survival and activation of inflammatory effector cells on the other side [17,82]. In our case, normal skin fibroblast cells exposed to AGEs-BSA responded promptly with increased GM-CSF levels, in a dose dependent manner (Fig 6). In our experimental model, AGEs-BSA exposure triggered signaling events leading to GM-CSF synthesis, which is an important contributor to perpetuated fibrotic and inflammatory reactions [82]. Surprisingly, TGF-β1 blockade diminished IL-6 and GM-CSF levels in AGEs milieu (Fig 8D and 8F), although TNF-α was additionally increased (Fig 8H). In fibroblasts, TGF-β1 was shown to induce TIMP-3 expression [83], an inhibitor of TNF-α converting enzyme [84], thus TGF-β1 negatively regulates TNF-α activation. These results indicate that TGF-β1 might have an inhibitory effect on IL-8 and TNF-α expression in normal fibroblasts (Fig 9), and by these means exert its anti-inflammatory role. TGF-β1 negative regulation of IL-8 was reported in neutrophils [37] and severe TNF-α and IFN-γ increases in TGF-β1 knock out mice resulted in wasting syndrome, immune cell infiltration and tissue necrosis [85].

The 200 μg/ml AGEs-BSA challenged fibroblasts had increased IFN-γ levels after both the 12 h and 24 h exposure intervals (Fig 6G). AGEs were previously shown to be inducers of both the IFN-γ pathway and several of its downstream effector molecules [86]. IFN-γ has a complex and dual role in inflammation and fibrosis. Classically, IFN-γ has been considered an inflammatory cytokine, yet several studies have shown IFN-γ possess immuno modulatory and protective properties, its administration being associated with improved prognosis in afflictions involving exacerbated fibrotic responses, such as systemic sclerosis [87,88] and chronic hepatitis C [89]. Diminished levels of IFN-γ were found in patients with idiopathic pulmonary fibrosis [90] and it has been suggested that the cytokine might be used for therapeutic purposes [91]. In our case, IFN-γ levels increased after exposure to 200 μg/ml AGEs-BSA and were unaffected by RAGE or TGF-β1 blockade, suggesting another AGEs-activated mechanism might be involved in its upregulation (Figs 8G and 9).

Notably, IL-10 expression was not detected in CCD-1070Sk fibroblasts. Recent findings have shown functional differences between fetal and adult fibroblasts essentially involve IL-10 cytokine, which is only expressed in the fetal phenotype [92]. The respective study also showed that IL-10 is associated with regenerative wound healing [92], suggesting IL-10 absence might be involved in fibrotic pathologies.

**Fig 10:**
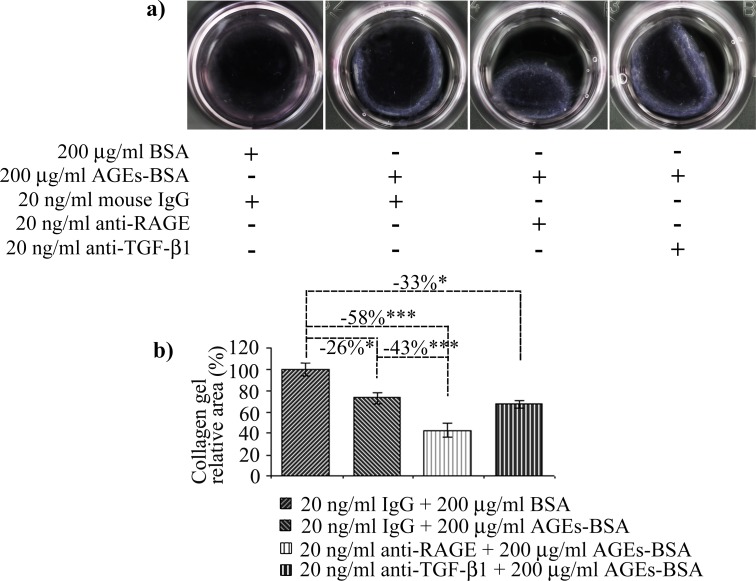
Collagen type I gel contraction by fibroblasts exposed to AGEs-BSA. Representative gels are shown in (a). Gel areas, reported to control (BSA and IgG treated cells) are shown in (b). Data represent means ± relative SD. Statistically significant changes are indicated * p< 0.05; ** p< 0.01; *** p< 0.001.

All the AGEs-BSA induced changes discussed above supported a contractile phenotype in CCD-1070Sk cells. As TGF-β1 is the central mediator of such transformation, we were surprised that TGF-β1 blockade did not inhibit collagen gel contraction. This outcome is most probably the result of the increased level of TGF-β1 gene expression we noted for 24 h cells exposed to 200 µg/ml AGEs-BSA, that could have contributed to increase the cytokine’s levels. Of particular interest in the effect of RAGE receptor blockade, which stimulated the contraction of collagen gels (Fig 10). It appears that AGEs-RAGE pro-inflammatory signaling inhibited fibroblast differentiation towards a contractile phenotype, associated with fibrosis. This would probably explain the delayed/impaired wound healing in diabetes patients, as RAGE constant stimulation might be inhibiting wound contraction. By inhibiting contraction, characteristic of fibrosis, RAGE acts in disfavor of fibrogenesis. Other studies have shown RAGE receptor negatively contributes to fibrosis development, as its depletion induced more severe fibrotic responses [93].

## Conclusions

RAGE, TGF-β1, collagen I and III gene and protein expression were upregulated after exposure to AGEs-BSA, while MMP-2 was activated. Fibroblasts chronic stimulation with AGEs-BSA induced TGF-β1 expression, however, TGF-β1 blockade experiments demonstrated that this cytokine was not associated with increased collagen I synthesis, as opposed to RAGE, who's blockade strongly inhibited collagen I gene and protein expression most likely through NF-κB. Contrary, collagen III gene and protein expression was strongly dependent on both TGF-β1 and RAGE.

AGEs-BSA induced upregulation of IL-8 and TNF-α pro-inflammatory cytokines was dependent on RAGE signaling and at the same time it was inhibited by TGF-β1, suggesting they might participate in an inhibitory cross-talk between TGF-β1 and RAGE signaling. Notwithstanding, RAGE and TGF-β1 signaling co-stimulated the expression of IL-2 and IL-4 anti-inflammatory cytokines and modulated MMP-2 activity and collagen III expression, probably involving an ERK1/2 mediated crosstalk. In normal fibroblasts exposed to AGEs-BSA, TGF-β1 alone is able to induce GM-CSF and IL-6, apparently without RAGE intervention. Moreover, IFN-γ is increased in AGEs-BSA milieu in a RAGE and TGF-β1 independent mechanism.

Taken together, our study raises the possibility that RAGE’s proinflammatory signaling component might dampen fibrogenesis by inhibiting fibroblast contraction, thus antagonizing AGEs-TGF-β1 effects, which encourage the contractile phenotype. However, RAGE can also be viewed as contributor to fibrosis development, as it is pivotal for collagen I synthesis stimulated by AGEs. Our study contributes to the understanding of AGEs induced skin complications associated with diabetes and accelerated aging.
